# Olefin metathesis in multiblock copolymer synthesis

**DOI:** 10.3762/bjoc.15.21

**Published:** 2019-01-24

**Authors:** Maria L Gringolts, Yulia I Denisova, Eugene Sh Finkelshtein, Yaroslav V Kudryavtsev

**Affiliations:** 1 Topchiev Institute of Petrochemical Synthesis, Russian Academy of Sciences, Leninsky prosp. 29, 119991 Moscow, Russia

**Keywords:** ADMET, macromolecular cross metathesis, multiblock copolymers, olefin metathesis, ROMP

## Abstract

Multiblock copolymers constitute a basis for an emerging class of nanomaterials that combine various functional properties with durability and enhanced mechanical characteristics. Our mini-review addresses synthetic approaches to the design of multiblock copolymers from unsaturated monomers and polymers using olefin metathesis reactions and other ways of chemical modification across double C=C bonds. The main techniques, actively developed during the last decade and discussed here, are the coupling of end-functionalized blocks, sequential ring-opening metathesis polymerization, and cross metathesis between unsaturated polymers, or macromolecular cross metathesis. The last topic attracts special interest due to its relative simplicity and broad opportunities to tailor the structure and hence the properties of the copolymer products. Whenever possible, we analyze the structure–property relations for multiblock copolymers and point to their possible practical applications.

## Introduction

Nowadays, olefin metathesis has become a well-established field of organic and polymer chemistry. The discovery of metallocarbene initiators that are capable of catalyzing metathesis polymerization in a living fashion turned it into a powerful tool of polymer design [1]. Hundreds of linear, comb-like, graft-, bottle-brush, ladder, and other homopolymers and copolymers were synthesized [2–7]. Block copolymers combining properties of two or more individual polymers in one material attract ongoing attention from both experimentalists and theoreticians due to their intrinsic tendency to self-assemble into diverse microstructures [8–11]. Technological applications of block copolymers cover lithography [12], photovoltaics [13], membranes [14] and many other areas [15]. Most of the research is devoted to diblock and triblock copolymers, whereas multiblock copolymer studies are still much less common [3–4,16–18]. Aside from more complicated synthesis and characterization of multiblock copolymers, for decades it was thought that any sequence disorder along polymer chains hinders their ordering [19] so that the only interesting are regular multiblock copolymers that can form structures with more than one periodicity [20]. Meanwhile, theoretical investigations [21–23] and computer simulations [24–27] gradually revealed the high potential of random multiblock copolymers with respect to self-assembly. In recent years, it was demonstrated that such polymers can be prepared with many of the available techniques, including polycondensation [28], chain-shuttling polymerization [29], copper-mediated radical polymerization [30–32], reversible addition–fragmentation chain transfer polymerization [33–34], and intermacromolecular reactions [35–37]. Though the properties of multiblock copolymers are far from being fully explored and understood, their applications already include adhesives, barrier materials, emulsifiers, impact modifiers, and materials for electronics, fuel cells, gene and drug delivery [8–9,15,38–40]. Compared with diblock and triblock copolymers, not to speak about polymer blends, multiblock copolymers often demonstrate superior mechanical properties, biocompatibility, biodegradability, compatibilizing ability, and tendency to form bicontinuous phases needed for ionic and molecular transport [8–10,41–45]. On the other side, they retain individual properties of their comonomers, which are usually averaged and therefore lost in fully random copolymers of similar composition [46–47].

In this mini-review we consider the approaches to multiblock copolymer syntheses via olefin metathesis reactions developed mainly over the past ten years. The following sections address the achievements and perspectives of three main techniques used for this purpose, namely, sequential ring-opening metathesis polymerization, coupling of end-functionalized blocks, and macromolecular cross metathesis.

## Review

### Synthesis by sequential ring-opening metathesis polymerization

**Scheme 1:**
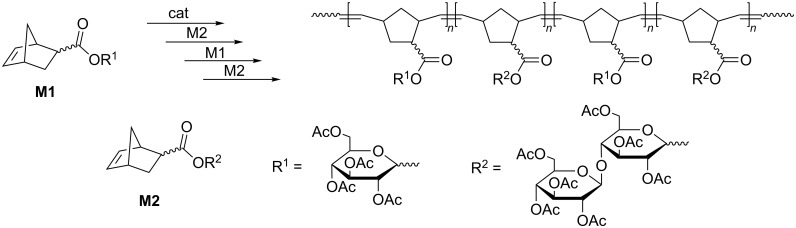
Multiblock copolymer synthesis by sequential ROMP, replotted from [51].

**Figure 1:**
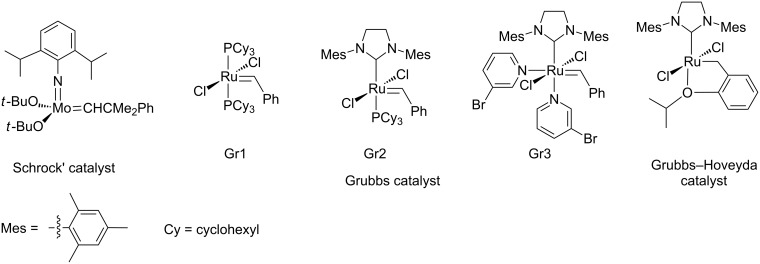
The most known commercially available catalysts for olefin metathesis.

Living ring-opening metathesis polymerization (ROMP) provides an opportunity to use a well-established route to multiblock copolymers based on the repetitive addition of different monomers to living polymer chains after full consumption of a previous monomer [48–49]. This technique was effectively applied for the synthesis of di-, tri- and tetrablock carbohydrate copolymers mediated by Schrock’ and Grubbs’ catalysts of the 1st (Gr1) and 2nd (Gr2) generations (Scheme 1, Figure 1) [50–51]. It yields copolymers of the desired average molecular mass and narrow molecular mass distribution (*Ð* = *M*_w_/*M*_n_ = 1.0–1.19) and enables control over the block sequence and length in the copolymer chains (sequence-controlled multiblock copolymers). However, in practice this method is restricted to copolymers with a limited number of blocks, such as tetrablocks or pentablocks [52], because each time a new monomer is added some of the living chains cannot initiate polymerization being terminated with trace impurities. Besides, in the course of ROMP main-chain double bonds are prone to secondary metathesis in a chain-transfer process that leads to reshuffling of the monomer unit sequences. Since less sterically encumbered groups are more easily involved into the secondary metathesis, this effect can be minimized by first polymerizing a more bulky monomer and then conducting a fast polymerization of another monomer [53–55].

### Synthesis from end-functionalized blocks

**Scheme 2:**
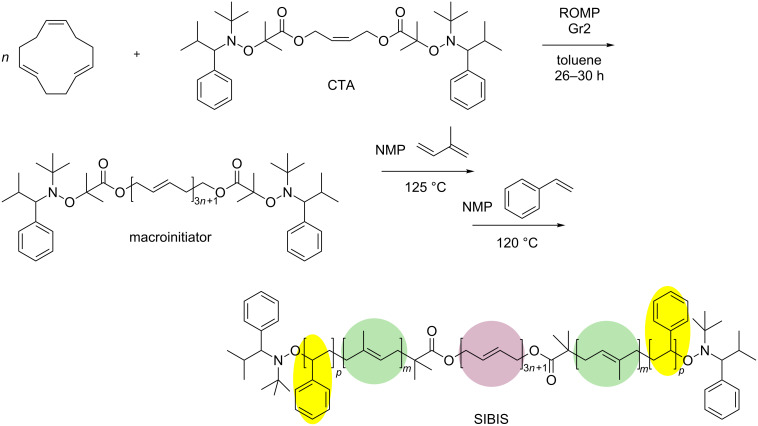
Multiblock copolymer synthesis by combining ROMP and NMP, replotted from [56].

Another strategy to multiblock copolymer preparation is to assemble them by using pre-synthesized telechelic polymers with α,ω-bifunctional end groups, which can be coupled in different ways. The classical technique for preparing telechelics uses a symmetrical difunctional olefin compound as a chain-transfer agent (CTA). This was applied for the synthesis of styrene (S)–isoprene (I)–butadiene (B) multiblock copolymers by combining ROMP with nitroxide-mediated polymerization (NMP) [56]. A perfectly regioregular α,ω-telechelic poly(1,4-butadiene) bearing alkoxyamine termini was obtained by ROMP of *trans*,*trans*,*cis*-1,5,9-cyclododecatriene in the presence of a symmetric acyclic olefin CTA (Scheme 2). This telechelic polybutadiene was used as the macroinitiator for the NMP of styrene and diene monomers to yield unimodal SBS, IBI, and SIBIS multiblock copolymers, which include glassy, rubbery, and semicrystalline polymer segments and demonstrate peculiar mechanical behavior [57–58].

**Figure 2:**
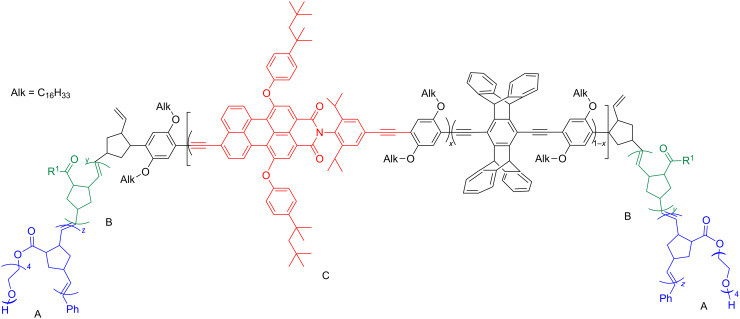
A highly fluorescent multiblock copolymer for bioimaging and in vivo tumor targeting [60].

References [59] and [60] report on the preparation of fluorescent polymer nanoparticles for bioimaging and in vivo targeting of tumors and the nanoparticles were formed by a ABCBA pentablock copolymer. In this polymer A stands for hydrophilic oligo(ethylene glycol) (OEG)-grafted polynorbornene possessing stealth-like and antifouling properties that are useful for in vivo applications. The B block is formed by polynorbornene functionalized with *N*-hydroxysuccinate esters (NHS) that can be used as a carrier for antitumor drugs, and the C block is a far-red emitting conjugated random copolymer of *p*-phenylene ethynylene (PPE) and perylene monoimide (PMI, Figure 2). For the synthesis, the random PPE–PMI copolymer was end-capped with norbornadiene (NB–(PPE–PMI)–NB) to allow further functionalization through olefin metathesis. The separately prepared by ROMP living diblock copolymers comprising norbornene with OEG (A block) and an NHS (B block) were synthesized in the presence of Gr1 and terminated by the reaction with NB–(PPE–PMI)–NB to obtain the ABCBA pentablock copolymers.

This copolymer forms nanoparticles with a central hydrophobic core capable of accommodating fluorescent dyes and conventional therapeutics and a hydrophilic biocompatible outer shell.

**Scheme 3:**
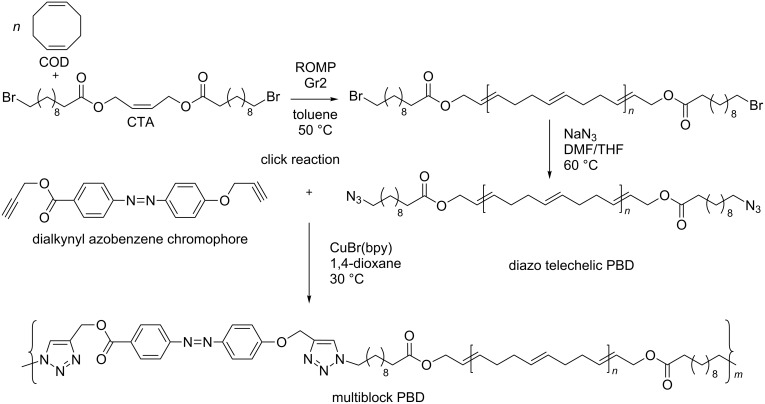
Multiblock copolymer synthesis by combining ROMP and click reactions replotted from [61].

The efficient combination of the ROMP process and click chemistry led to the highly photoresponsive multiblock polybutadiene [61]. Initially, ROMP of 1,5-cyclooctadiene (COD) in the presence of a difunctional CTA provided dibromo-telechelic polybutadiene (PBD), which was transformed into diazido-functionalized telechelic PBD (Scheme 3).

The multiblock PBD then was assembled by multiple click reactions of the diazido-telechelic PBD with a dialkynyl-containing azobenzene chromophore. The newly formed triazole moieties can tune and improve the photoresponsive properties of PBD.

**Scheme 4:**
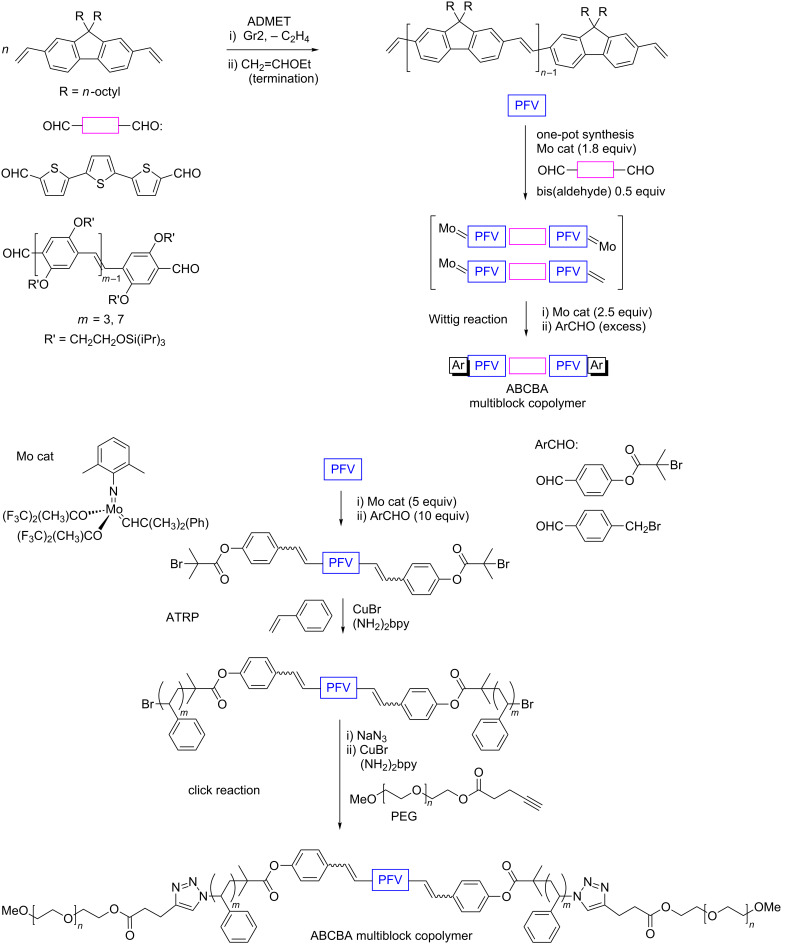
Multiblock copolymer synthesis by combining ADMET and other reactions, replotted from [63–64].

α,ω-Functional telechelic polymers also can be synthesized by acyclic diene metathesis (ADMET) polymerization. This approach was implemented for the preparation of fluorene-containing multiblock copolymers [62–63]. Poly(9,9-di-*n*-octylfluorene-2,7-vinylene, PFV) obtained by ADMET polymerization of 2,7-divinyl-9,9-di-*n*-octylfluorene in the presence of Gr2 under reduced pressure (Scheme 4), possessed exclusive *trans* regularity and contained vinyl groups at the both polymer chain ends. These groups were treated with a Mo catalyst to generate the corresponding Mo-alkylidene moieties followed by the Wittig-type cleavage with various aldehydes, gave an opportunity to utilize atom transfer radical polymerization (ATRP) and сlick reactions for the precise synthesis of amphiphilic ABCBA-type block copolymers (Scheme 4) [63]. A more facile “one-pot” procedure for the synthesis of an end-functionalized conjugated multiblock copolymer with PFV main chain was accomplished by combining olefin metathesis and subsequent Wittig coupling (Scheme 4) [64].

The ADMET technique was used not only for the synthesis of polymer telechelics but also for their assembling into multiblock copolymers. A simple one-pot way for the preparation of random multiblock copolymers was proposed in reference [65]. A mixture of semicrystalline and amorphous samples of partly hydrogenated PBD underwent ethenolysis in the presence of the Ru-carbene catalyst. This depolymerization procedure resulted in the formation of telechelics with both end vinylated. Then, the ethylene atmosphere was replaced with argon and an additional amount of catalyst added. Under these conditions, the ADMET polymerization led to the multiblock copolymers with randomly distributed semicrystalline and amorphous blocks, which exhibited noticeably improved mechanical properties compared with the blend of the initial polymers.

**Scheme 5:**
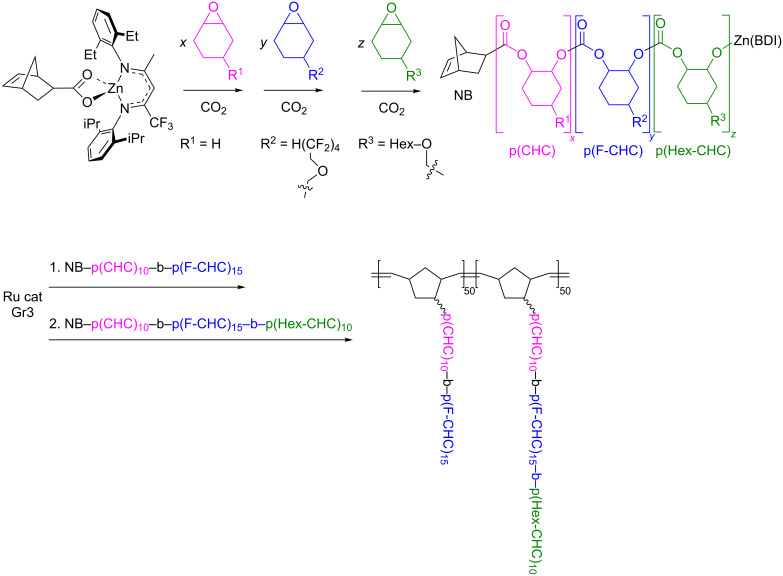
Synthesis of multiblock bottle-brush copolymers by ROMP, replotted from [68].

An approach utilizing macromonomers or macrocycles was used for the synthesis of multiblock copolymers with random or sequence-controlled structure [66]. The ROMP is also suitable for the synthesis of bottle-brush block copolymers, in which linear or branched side chains are densely grafted to a linear backbone, being easily functionalized for recognition, imaging, and drug delivery in aqueous media [4–6,67]. They have a low tendency to entangle and can rapidly self-assemble in selective solvents even at very low concentrations forming large-domain microstructures. The facile synthesis of norbornenyl-terminated di- and triblock poly(cyclohexene carbonate)s was carried out by the β-diiminate (BDI) zinc-catalyzed block copolymerization of functionalized epoxides and CO_2_ with a norbornenyl-containing initiator (Scheme 5) [68]. The subsequent “grafting through” by ROMP of norbornene resulted in the synthesis of multiblock copolymer brushes. Changes in the synthetic stage sequence led to variable layer compositions.

**Scheme 6:**
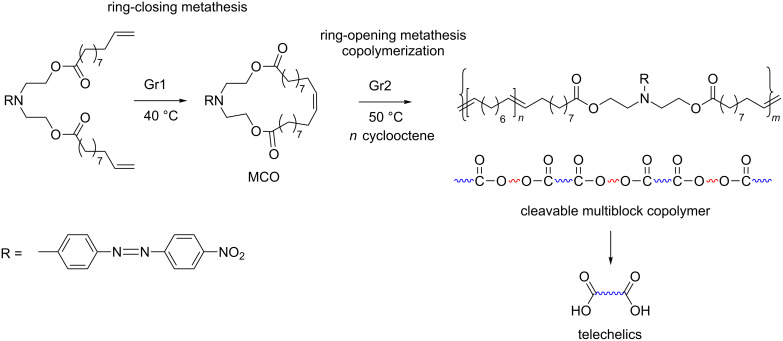
Sacrificial synthesis of multiblock copolymers, replotted from [70].

Various linear and star-shaped (triarm) ABA and ABCBA amphiphilic multiblock copolymers containing acetal-protected sugars (APS) were prepared by the coupling of an end-functionalized ROMP copolymer of norbornene (NB) and APS-substituted NB with poly(ethylene glycol) (PEG) [69]. Ring-opening metathesis copolymerization of the rather strained cyclooctene (COE) and a strainless 27-membered macrocyclic olefin (MCO) led to the multiblock copolymer consisting of octenylene blocks linked with ring-opened MCO segments (Scheme 6) [70]. The higher reactivity of COE in ROMP is the reason for the formation of long octenylene sequences.

The MCO was obtained by ring-closing metathesis and contained easily cleavable ester linkages. It gave the possibility to сut the multiblock copolymer into pieces under alkaline conditions in order to obtain telechelic polyoctenylene with carboxyl end groups. The last reaction represents an example of the so-called sacrificial synthesis, another effective approach to telechelics [71].

Hiff and Kilbinger generated cleavable ABAB pentablock and ABABABA heptablock metathesis copolymers via the sequential ROMP of seven-membered cyclic acetals (2-methyl-1,3-dioxepine and 2-phenyl-1,3-dioxepine) and N-substituted NB dicarboximide derivatives [72]. The subsequent hydrolysis of the prepared copolymers resulted in well-defined telechelics in good yields per initiator molecule and thus significantly improved the initiator efficiency. The sacrificial approach also helps to describe the multiblock copolymer structure: owing to the acid-labile acetal group, polymer scission takes place at the point of the dioxepin insertion thus providing an indirect way to detect the monomer location [73].

**Scheme 7:**
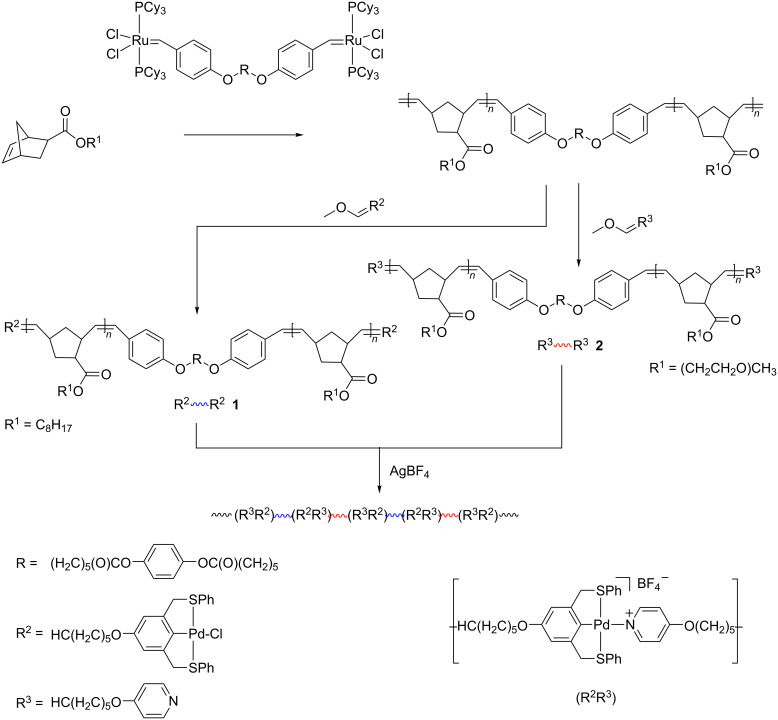
Synthesis of supramolecular multiblock copolymers, replotted from [74].

Supramolecular multiblock copolymers with the possibility to introduce stimuli-responsive functionalities were obtained using a bimetallic ruthenium initiator [74]. The initiator allowed for the single-step fabrication of symmetrically end-functionalized telechelic polymers using ROMP and functional chain terminators (Scheme 7). In more detail, the synthesis included ROMP of NB octyl ester or NB by means of metal coordination using the obtained telechelic polymers methyl triglycol ester in the presence of the bimetallic ruthenium catalyst followed by the addition of an excess of either a Pd-containing chain terminator to obtain pincer-functionalized telechelic polymer **1** or a pyridine-containing end-terminator to yield pyridine-functionalized telechelic polymers **2**. On this basis, supramolecular copolymers with alternating blocks were constructed using AgBF_4_ to remove Cl from the pincer complex and generate a cationic Pd ligand, which can coordinate with pyridyl ligands in a new pincer complex.

**Figure 3:**
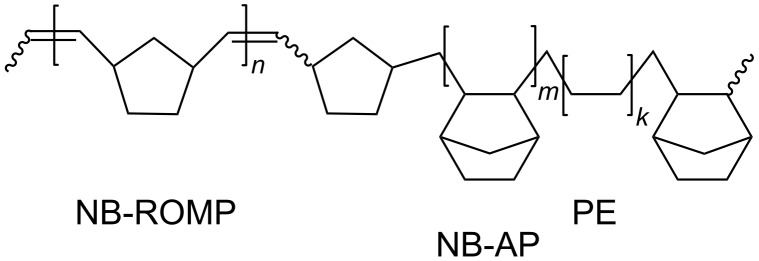
The multiblock copolymer capable of post-functionalization [76].

A range of Zr(IV) and Hf(IV)-based bisamido complexes can catalyze both ROMP and addition (AP or vinyl) (co)polymerization of NB [74–75]. The presence of a 2-pyridyl moiety, along with a boron-containing group, and activation by MAO makes it possible to synthesize a NB copolymer with ethylene, containing both NB–ROMP and NB–AP monomer units. This approach allows obtaining multiblock copolymers that are capable of simple post-polymerization functionalization across double bonds (Figure 3). For instance, the introduction of polar groups imparts adhesive properties to the copolymers, which are essential for coatings.

### Synthesis by macromolecular cross metathesis

**Figure 4:**
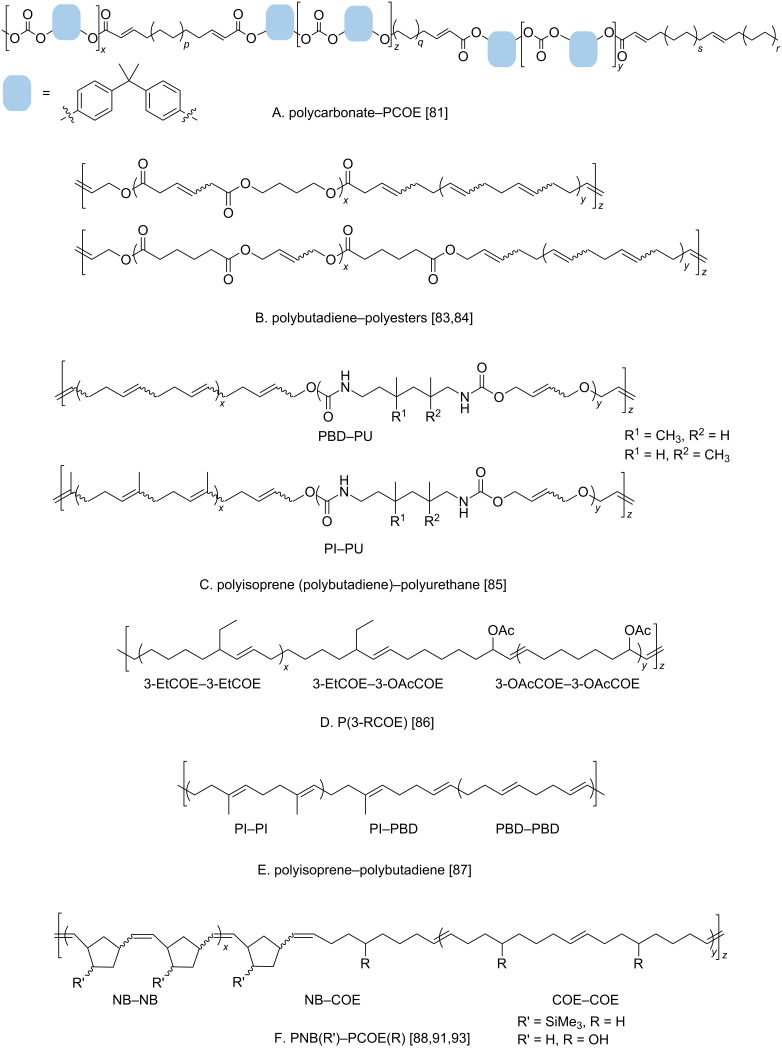
Multiblock copolymers synthesized by macromolecular cross metathesis.

Cross metathesis between polymers containing main-chain C=C double bonds is a recent and actively developing approach to random multiblock copolymers. For years, the cross metathesis involving double bonds in the polymer backbone was considered as an undesired chain-transfer process that broadens molecular mass distribution, leads to the formation of cyclooligomers and reshuffling of monomer units in the course of the polymer synthesis [77]. The cross metathesis reactions on polymers were mostly studied with regard to the intramolecular polymer–catalyst interactions [77–79] or intermolecular degradation of polymers via the cometathesis with different olefins [77,80–81]. Only recently, the cross metathesis between macromolecules, or macromolecular cross metathesis (MCM), began to be considered as a promising reaction for various applications [82–93]. It was shown that the random copolymers produced by the cross metathesis of chemically dissimilar polymers, such as polycarbonate and PCOE, demonstrate an ability to ordering via microphase separation (Figure 4A) [82]. The MCM was shown to be effective in the preparation of multiblock copolymers from parent polymers synthesized according to different polymerization mechanisms. New multiblock copolymers were obtained by the cross metathesis of ROMP-derived 1,4-polybutadiene or natural polyisoprene and olefin-containing polyester or polyurethane prepared via step-growth polymerization (Figure 4B and C) [83–85]. The multiblock copolymers from polybutadiene and olefin-containing polyurethane demonstrated improved mechanical properties [85]. Head-to-tail regioregular and *E*-stereoregular multiblock copolymers and heterotelechelic polymers were successfully synthesized by the cross metathesis between different ROMP-derived poly(3-substituted cyclooctenes), (Figure 4D) [86]. The MCM between immiscible commercial polybutadiene and polyisoprene led to the formation of single-phase block copolymers (Figure 4E) [87]. The cross metathesis between functionalized polyoctenamers (PCOE) and polynorbornenes (PNB) opened the way to new multiblock copolymers that are difficult to obtain by other methods (Figure 4F) [88–93]. With a large excess of COE, the ring-opening metathesis copolymerization of NB and COE results in the formation of a mixture of the homopolymers and copolymers enriched with NB units [94–95]. The substantial difference in the monomer strain energy (NB: 100 kJ mol^−1^, *−*Δ*G°* ROMP = 47 kJ mol^−1^; COE: 16 kJ mol^−1^, *−*Δ*G°* ROMP = 13 kJ mol^−1^) [77,96] is the reason for such behavior. Unlike copolymerization, the MCM starts from two homopolymers, PNB and PCOE, in which there is no difference in the strain energy, so that multiblock copolymers with various block lengths are easily formed [88–89,91,93]. Obtaining of multiblock polymers using cross metathesis is synthetically much simpler than using the earlier described sequential ROMP or pre-synthesized block-coupling techniques so that MCM can be advantageous when a strict sequence control over the copolymer structure is not needed. Nevertheless, random block copolymers obtained by interchain exchange reactions, like MCM, retain the ability to ordering [82].

**Scheme 8:**
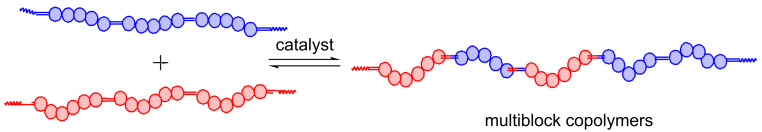
Macromolecular cross metathesis.

MCM is an interchain cross reaction characterized by reshuffling of monomer units in the macromolecular backbones via break up and formation of new double bonds according to the olefin metathesis mechanism. In the beginning, an exchange of chain segments between the parent homopolymers results in the formation of diblock copolymers. Then random multiblock copolymers are formed (Scheme 8), their average block lengths are decreased until they gradually reach the values typical of a copolymer with the fully random unit sequence.

**Figure 5:**
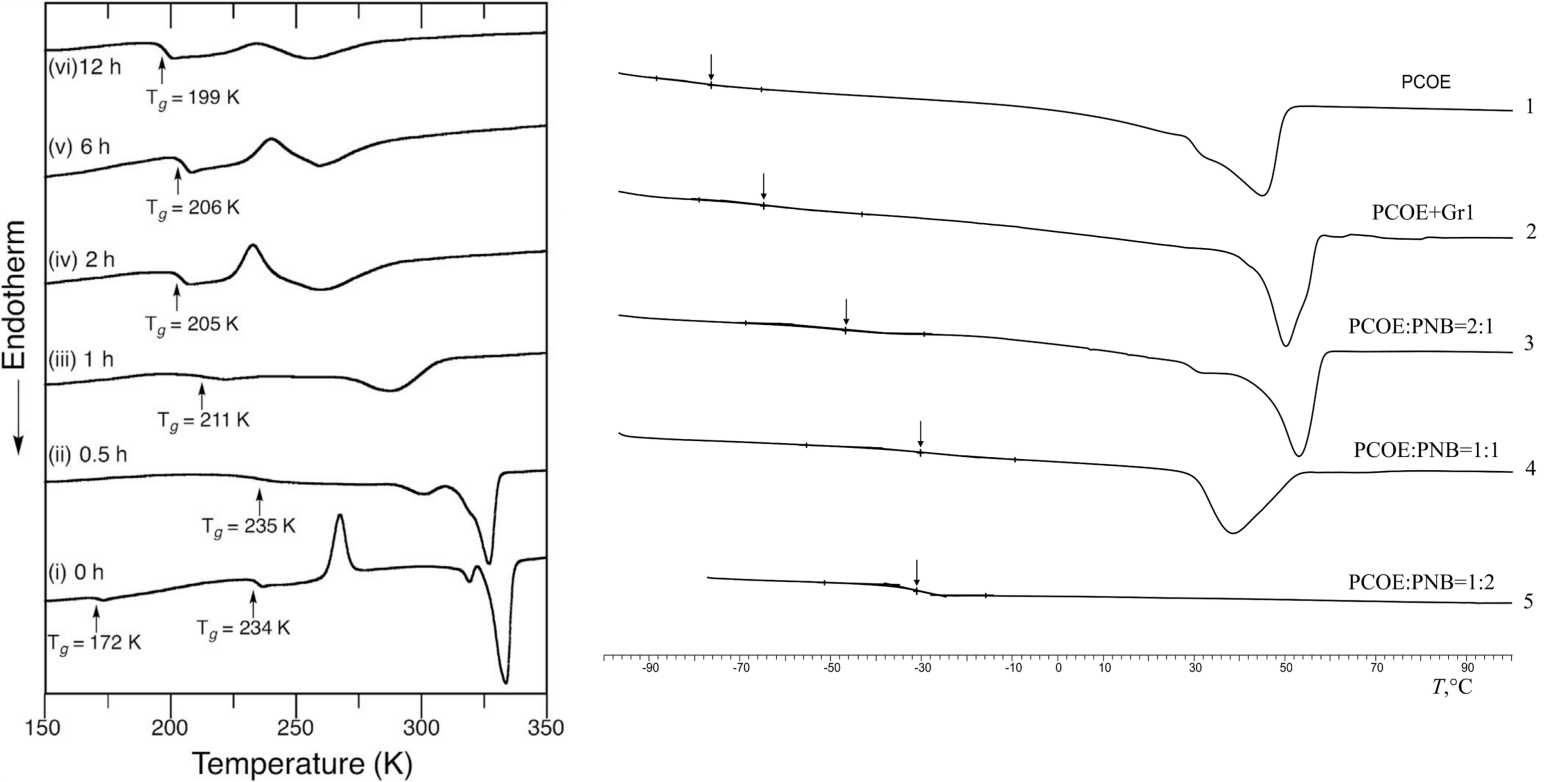
Changes in the DSC thermograms during MCM of PBD and polyesters (left) [84] and PNB–PCOE (right) mediated by Gr1 catalyst [89]. Arrows indicate the glass transition temperatures.

Therefore, the copolymer chain structure can be controlled by altering the reaction time, molar ratio of the starting polymers, catalyst type and concentration, as well as solvent type and initial polymer concentration [83–89,93]. It is important to keep a relatively high polymer concentration in the reaction mixture to prevent intramolecular metathesis that leads to cyclooligomers. It is worth noting that the Gr2, Gr3 and Grubbs–Hoveyda (Gr–H) catalysts (Figure 1) are much more active than Gr1 in MCM and concentrations of 0.036–0.049% are sufficient to carry out the process effectively [86–87]. In the PBD–polyisoprene (PI) cross metathesis, Gr1 can be replaced by Gr–H1 but longer reaction times are needed [87]. A control over the reaction kinetics can be sometimes complicated because the overall composition of a polymer mixture does not change in the course of MCM. Nevertheless, it can be successfully implemented using a complex of NMR, GPC, and DSC methods. As a rule, the parent polymers are characterized by different molecular masses, which allow using GPC to track how two peaks in the chromatogram merge into one with conversion. If the initial polymers display different glass transition temperatures, DSC can be also used to monitor the kinetics (Figure 5). At the beginning of the MCM reaction, two *T*_g_ values are observed which get closer and finally merge into one, when long sequences of chemically identical units stemming from the parent homopolymers are exhausted.

**Figure 6:**
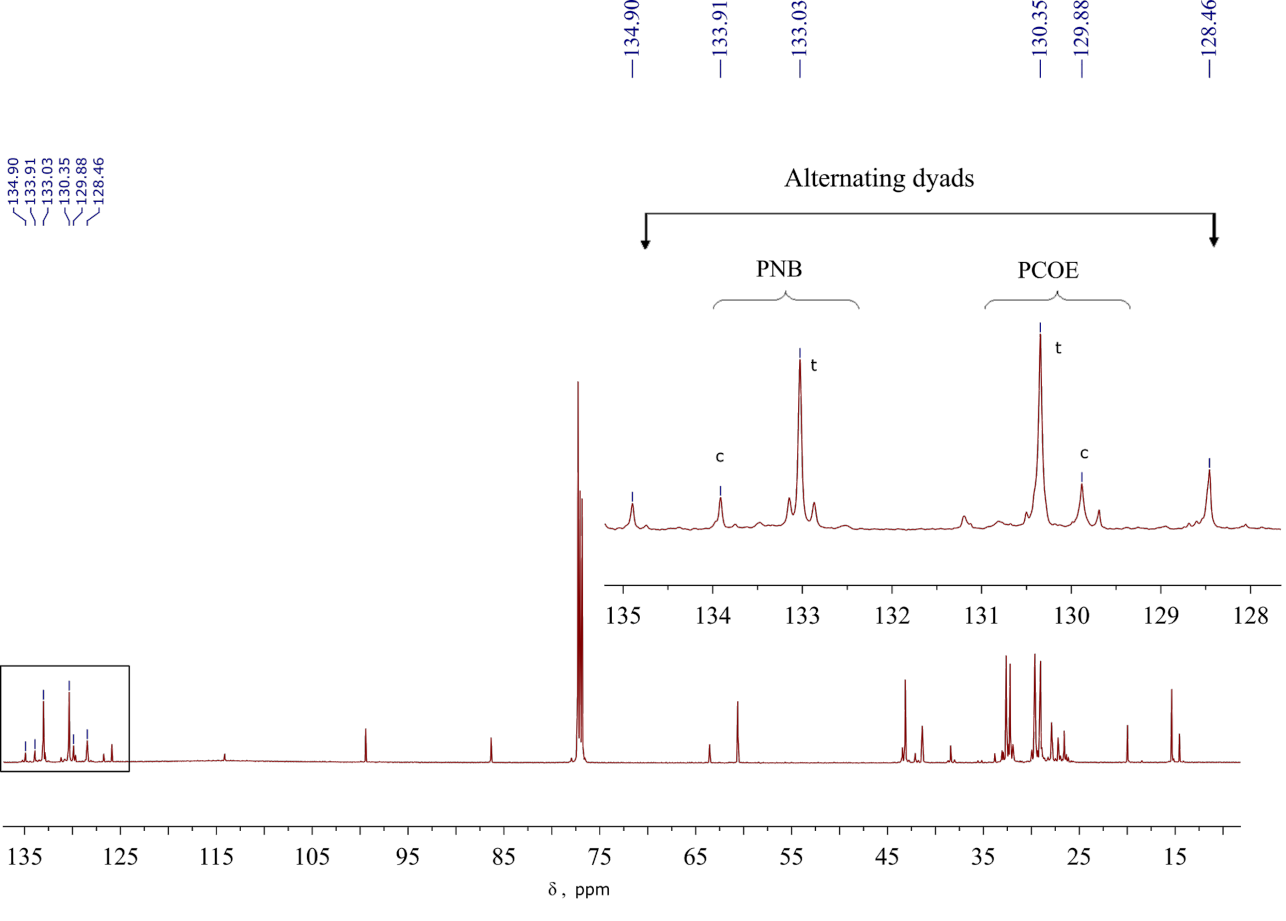
The ^13^C NMR spectrum recorded after 8 h of the reaction between PCOE, PNB, and Gr1; the homo- and heterodyad signals are enlarged in the inset [90].

^1^H NMR spectroscopy was implemented to track the evolution of the chain structure in the course of MCM between polybutadiene (PBD) or polyisoprene (PI) and olefin-containing polyesters or polyurethane, as well as changes in the chain stereospecificity during the reaction between 3-substituted PCOEs [83–86]. Cross metathesis in the PNB/PCOE (Figure 6) and PBD/PI pairs was monitored by ^13^C NMR [87–91,93]. The fraction of heterodyads in the copolymer gradually increased with conversion thus indicating the formation of random multiblocks. The average block length *L* was calculated from an integral ratio of homo (A–A, B–B) and heterodyad (A–B) signals in the NMR spectra:

*L*_A_ = [*I*(*C*^A–A^)*+I*(*C*^A–B^)]/*I*(*C*^A–B^); *L*_B_* =* [*I*(*C*^B–B^)+*I*(*C*^B–A^)]/*I*(*C*^B–A^*)*; where *I*(*C*^A–A^) and *I*(*C*^B–B^) are the peak intensities of the initial homodyads, A–A and B–B, and *I*(*C*^A–B^) and *I*(*C*^B–A^) are the peak intensities related to the alternating dyads.

The average block lengths decreased with the conversion, reaction time, and catalyst concentration and asymptotically approached the value of 2, characteristic of a completely random (Bernoullian) equimolar copolymer. Thus, a proper choice of the MCM conditions enables one to obtain copolymers with a controllable average block length ranging from the initial homopolymer length to a few monomer units.

**Scheme 9:**
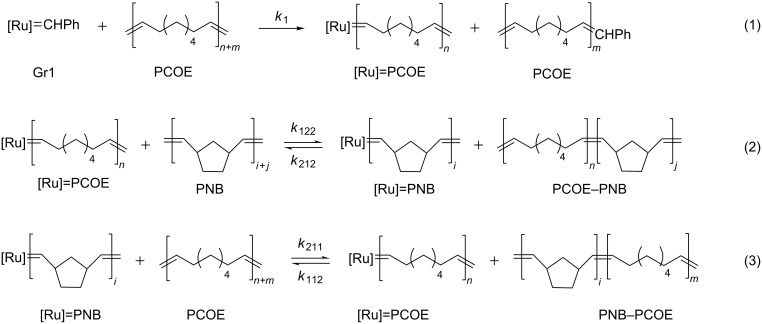
Elementary reactions of MCM between PNB and PCOE, replotted from [90].

**Figure 7:**
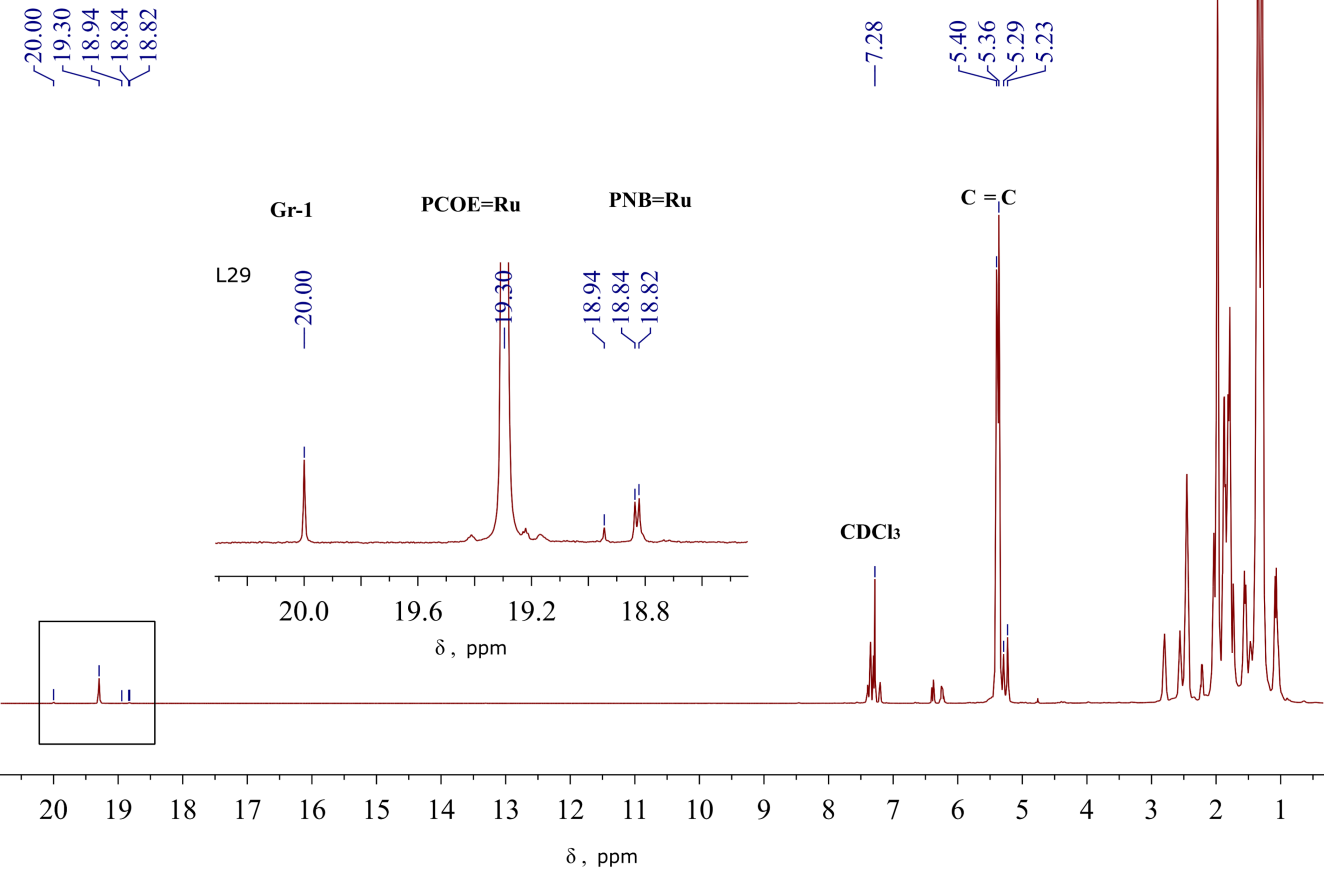
The ^1^H NMR spectrum recorded after 24 h of the reaction between PCOE, PNB, and Gr1 in CDCl_3_. The carbene signals are enlarged in the inset [90].

Important data on the kinetics of MCM between PNB and PCOE mediated by Gr1 were obtained by combining in situ NMR studies of the Ru-carbene transformations and ex situ NMR monitoring of the dyad composition evolution [90]. It was found that Gr1 first interacts with PCOE so that all Ru-carbenes become bound to those macromolecules approximately within one hour (Scheme 9, reaction 1 and Figure 7). Recall that the addition of Gr1 to a mixture of NB and COE first causes rapid metathesis polymerization of NB and only after that COE monomers are involved. An early MCM stage is also characterized by a decrease in the average molar mass of the mixture, which indicates that polymer backbones break during their interaction with the catalyst.

It takes about a day for the interchain exchange between the homopolymers with carbene-functionalized end groups to yield a statistical NB–COE copolymer and during this process its molar mass remains almost unchanged. The slowest elementary reaction, which controls the overall kinetics, is the interaction between [Ru]=PCOE carbenes and C=C bonds in PNB chains (Scheme 9, reaction 2). Its low rate is consistent with the bulky structure of NB units. During the cross metathesis, the concentration of [Ru]=PNB carbenes is very low but they are necessary for the cross reaction to proceed (Scheme 9, reaction 3).

An increase of the PNB concentration in the mixture results in a growth of the copolymer degree of blockiness [89]. This feature of the cross metathesis between PNB and PCOE is also opposite to what is expected for the metathesis copolymerization of NB and COE, where a high excess of COE is needed to allow for the formation of NB–COE copolymer [95].

Some results regarding the *cis*/*trans*-isomerization of double bonds in the MCM process were obtained [85,87,89]. In the systems PNB–PCOE (68% *cis*)–Gr1 and PBD–*cis*-olefin-containing polyurethane (*cis-*PU)–Gr2, *cis*-double bonds partly transform to a more thermodynamically stable *trans*-configuration, which is well-known for olefin metathesis [85,89]. The *cis*/*trans*-isomerization is observed for homodyads in MCM and even in the course of the homopolymer–catalyst interaction as a result of self-metathesis reactions that do not directly influence the copolymer formation. For instance, the cross metathesis of commercial *cis*-PBD (97% *cis*, 2% *trans*, 1% vinyl) with *cis*-PI (94.5% *cis*, 5.5% *trans*) mediated by Gr1 led to a partial conversion of *cis*-double bonds in PBD units into the *trans*-configuration increasing its content from 2 to 9% [87]. On the opposite, the amount of *trans*-double bonds in PI decreased, which resulted in the increase of the *cis*-double bonds content from 94.5 to 99%. The authors explained this observation by the higher reactivity of isoprene *trans* units. However, the *cis*-PU was more active in the MCM reaction with PBD than *trans*-PU [85]. It seems that more research on this topic is needed. It is also worth mentioning that the MCM of 3-substituted PCOEs proceeds in a regioselective fashion, similar to the ROMP of 3-substituted COE monomers [86].

Choosing a suitable solvent is of vital importance for the effective implementation of MCM reactions. It should provide homogeneity of the reaction medium at a highest possible polymer concentration to minimize the impact of intrachain reactions [79]. At the same time increasing polymer concentration can lead to polymer/solvent and polymer/polymer phase separation. These issues can be controlled by light scattering [90]. Another possible concern is related to the high viscosity of the initial polymer mixture, especially in the case of high molecular mass components, like PNB. Fortunately, upon the catalyst addition such mixtures rapidly become more fluid because of polymer-chain scission. The effect of solvent (THF and CH_2_Cl_2_) was studied for the MCM in the PBD–PU–Gr2 system [85] and it was found that the reaction in THF proceeded at a higher rate than in CH_2_Cl_2._

A decrease in the polymer molecular mass can be considered as a disadvantage of the MCM process. It takes place at the first stage of the reaction when Ru–polymer carbene active sites are formed as a result of the catalyst–polymer interaction. The decrease in *M*_n_ is observed during the first 1–2 hours and then it remains nearly unchanged [84]. The molecular mass of the resulting multiblock copolymer decreases with increasing the catalyst concentration [84,88]. Another reason for lowering the copolymer molecular mass is related to intramolecular metathesis that leads to low molecular mass cyclooligomers [77], which are lost during isolation of the reaction product. This negative effect can be partially counteracted by increasing the polymer concentration in the reaction mixture [84] in order to suppress intramolecular reactions.

**Scheme 10:**
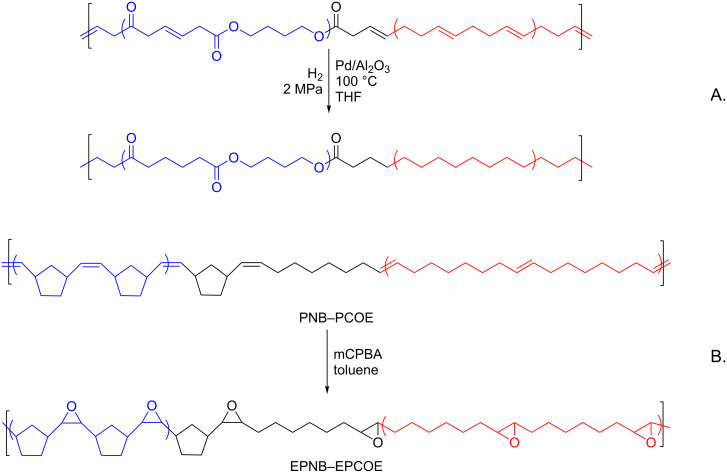
Post-modification of multiblock copolymers by hydrogenation (A) [85] and epoxidation (B) [101] of C=C double bonds.

The range of practical applications of multiblock copolymers can be significantly broadened through their functionalization. This goal can be achieved by introducing substituents into the parent homopolymers before MCM, just to mention 3-substituted PCOEs that are able to form stereoregular structures [86]. We introduced substituents into NB–COE copolymers by premodification of NB blocks or COE blocks (Figure 4F) [91,93]. A bulky Me_3_Si-substituent that can enhance gas separation properties was introduced into NB copolymer blocks by the cross metathesis of poly(5-trimethylsilylnorbornene) with PCOE [89]. Kinetic studies demonstrated that a substituent in the NB monomer units considerably lowers the MCM rate. The introduction of hydroxy groups into COE units of a NB–COE copolymer met certain difficulties mainly related to the poor solubility of the parent poly(5-hydroxycyclooct-2-ene), PCOE(OH), homopolymer in common solvents [91,97]. The cross metathesis of PNB with PCOE(OH) in the presence of Gr2 was carried out only in a mixed solvent, CHCl_3_ (10%)/MeOH. However, MCM was accompanied by partial hydrogenation of double bonds, especially for long reaction times. The ability of the Gr2 catalyst to form Ru–hydride complexes in the presence of alcohols is well-known and described in the literature [98–99]. Such complexes promoting C=C bond hydrogenation were detected in the PNB–PCOE(OH)–Gr2 system using NMR [97]. It is curious that the resulting multiblock copolymers reveal some crystallinity, whereas the parent PNB and PCOE(OH) are fully amorphous. It can be explained if we recall that hydrogenated PNB is a semicrystalline polymer [100]. The Pd/Al_2_O_3_ catalyst was used to promote the hydrogenation of multiblock copolymers formed with the cross metathesis of PBD and olefin-containing polyester (Scheme 10A) [84]. It was shown that shortening the block length in both the olefinically unsaturated and hydrogenated copolymers resulted in a decrease, and, finally, in the extinction of *T*_m_. At the same time multiblock copolymers with long blocks demonstrated two glass temperatures, which get closer to each other upon block shortening and then a single-phase copolymer with one *T*_g_ was formed [84,86–88]. Besides, the semitransparent, hard, and brittle copolymers obtained by MCM of PBD and polyesters became nearly transparent and flexible upon hydrogenation [84]. Another approach to the post-functionalization of NB–COE multiblock copolymers was implemented in reference [101] via double-bond epoxidation in the presence of *m*-chloroperbenzoic acid (Scheme 10B). It was found that this reaction proceeds more actively in the COE copolymer blocks than in the parent PCOE homopolymer. The epoxidation, as well hydrogenation, influenced the thermal and crystalline properties of the multiblock copolymers resulting in the increase of *T*_g_ by 40–50 °С and *T*_m_ by 20–30 °C. It is quite natural that the degree of crystallinity and melting temperature are higher for the copolymers with longer COE blocks.

**Figure 8:**
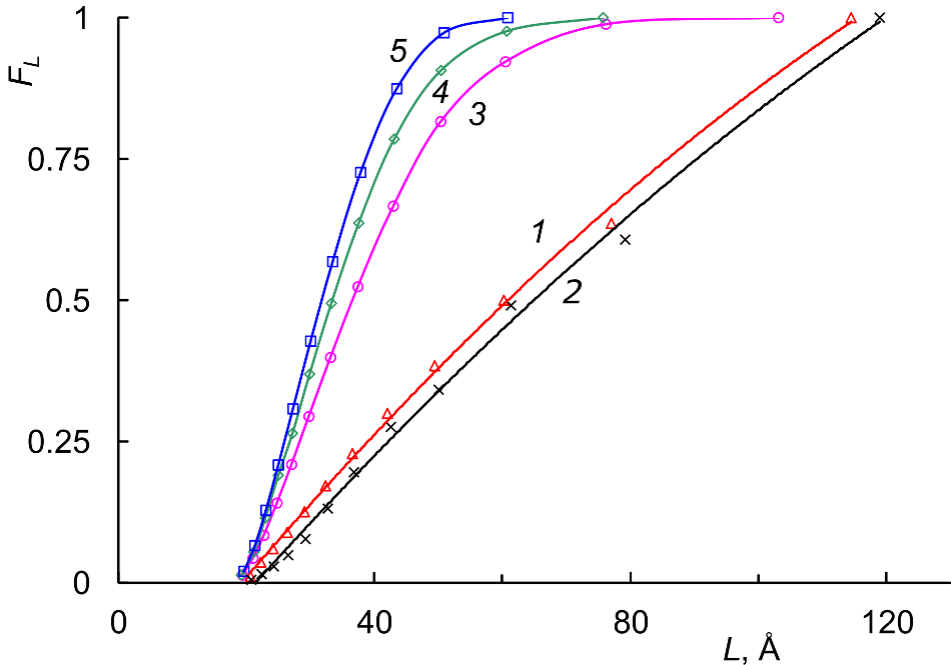
Integral distribution functions for the lamella thickness of crystallites in thermally fractionated (1) PCOE after Gr1 treatment, (2) PCOE/PNB blend, and NB–COE copolymers with different COE-block lengths, (3) *L*_COE_ = 22, (4) *L*_COE_ = 12, and (5) *L*_COE_ = 9.4 [92]

Copolymer crystallinity can be studied in detail by combining WAXD and DSC methods, including recently emerged technique of thermal fractionation by successive self-nucleation and annealing [84,92,102–103]. It was found that the width distribution of crystalline lamellae in NB–COE copolymers correlates with the average length of the *trans*-octenylene blocks. Compared with the pure PCOE or its equimolar blend with PNB, the NB–COE copolymers form considerably smaller crystallites (Figure 8) [92].

## Conclusion

It is rather clear nowadays that the olefin-metathesis reaction is a versatile tool for the synthesis of multiblock copolymers with diverse chemical structures. Due to the rapid progress in the catalyst design for living polymerization, sequential ROMP has become a well-established method of obtaining copolymers with sequence-defined structures. However, in many aspects, this technique remains laborious and even cumbersome. Most publications report on the multiblock copolymers synthesis by the coupling of premade individual blocks. A key point here is related to advances in the development of synthetic approaches for fabricating symmetric and asymmetric telechelics and monochelics, macromonomers and macrocycles based on different olefin-metathesis techniques like CTA, ADMET, etc. A subsequent assembling of macroblocks into copolymers can be carried out by combining olefin metathesis with other reactions such as ATRP, RAFT, click-reaction, and so on, which permit to gain certain control over the final copolymer structures. The most recent approach to the multiblock copolymer synthesis implements the macromolecular cross-metathesis reaction, which is still poorly studied. For this method, the simplicity of realization is counterweighted by inability of precise control over block sequences and considerable drop in the average molecular mass of reacting polymers as a result of their interaction with metathesis catalysts. Nevertheless, the average block lengths can be easily tailored and the resulting copolymers reveal the ability to self-assemble into ordered structures, enhanced mechanical properties, and nontrivial crystalline and thermal characteristics. Recent kinetic studies with the use of in situ and ex situ NMR have shed some light on the regularities of the macromolecular cross-metathesis reaction, which appeared to be somewhat opposite to the notions about metathesis copolymerization. Perspectives of the entire field under review are related to the elaboration of novel post-modification methods for obtaining new functionalities and enhancing various characteristics of multiblock copolymers. In our opinion, further development of the olefin metathesis methods for the multiblock copolymer synthesis will be directed by the search for new properties and possible applications.
